# Mesothelin and TGF-α predict pancreatic cancer cell sensitivity to EGFR inhibitors and effective combination treatment with trametinib

**DOI:** 10.1371/journal.pone.0213294

**Published:** 2019-03-28

**Authors:** Ethan Poteet, Dongliang Liu, Zhengdong Liang, George Van Buren, Changyi Chen, Qizhi Yao

**Affiliations:** 1 Michael E. DeBakey Department of Surgery, Division of Surgical Research, Baylor College of Medicine, Houston, Texas, United States of America; 2 Center for Translational Research on Inflammatory Diseases (CTRID), Michael E. DeBakey VA Medical Center, Houston, Texas, United States of America; University of South Alabama Mitchell Cancer Institute, UNITED STATES

## Abstract

Clinical trials of EGFR inhibitors in combination with gemcitabine for the treatment of pancreatic ductal adenocarcinoma (PDAC) have generated mixed results partially due to the poorly defined effectiveness of EGFR inhibitors in PDAC. Here, we studied a panel of PDAC cell lines to compare the IC50s of the EGFR inhibitors gefitinib and cetuximab. We found that gefitinib induced biphasic inhibition in over 50% of PDAC cells, with the initial growth inhibition occurring at nanomolar concentrations and a second growth inhibition occurring outside the clinical range. In contrast to gefitinib, cetuximab produced a single phase growth inhibition in a subset of PDAC cells. Using this sensitivity data, we screened for correlations between cell morphology proteins and EGFR ligands to EGFR inhibitor sensitivity, and found that mesothelin and the EGFR ligand TGF-α have a strong correlation to gefitinib and cetuximab sensitivity. Analysis of downstream signaling pathways indicated that plc-γ1 and c-myc were consistently inhibited by EGFR inhibitor treatment in sensitive cell lines. While an inconsistent additive effect was observed with either cetuximab or gefitinib in combination with gemcitabine, the cell pathway data indicated consistent ERK activation, leading us to pursue EGFR inhibitors in combination with trametinib, a MEK1/2 inhibitor. Both cetuximab and gefitinib in combination with trametinib produced an additive effect in all EGFR sensitive cell lines. Our results indicate that mesothelin and TGF-α can predict PDAC sensitivity to EGFR inhibitors and a combination of EGFR inhibitors with trametinib could be a novel effective treatment for PDAC.

## Introduction

The epidermal growth factor receptor (EGFR; ErbB-1) is a transmembrane tyrosine kinase receptor capable of regulating a diverse array of cellular functions including growth, survival, cell migration, and differentiation [1]. Activation of EGFR and its downstream pathways is a significant event in the formation of pancreatic ductal adenocarcinoma (PDAC), reinforcing ERK pathway activation to promote tumor transformation after the initial driver mutation(s) occur in KRAS [2]. Although the ERK pathway is a primary driver of PDAC, EGFR also signals through the STAT, Akt/PI3K, and PLC-γ pathways, and can directly translocate to the nucleus to activate c-myc and cyclin-D1 [3,4]. The utility of EGFR inhibition is disease dependent, with distinct pathways affected in different diseases. Identifying the specific pathways that are inhibited is critical for assessing the effectiveness of EGFR inhibition in PDAC.

Two classes of EGFR inhibitors have been developed for cancer therapy: 1) reversible tyrosine kinase inhibitors, including gefitinib and erlotinib; and 2) EGFR blocking antibodies, including cetuximab and panitumumab [5]. Previous studies on PDAC cells have reported a wide range of effectiveness to EGFR tyrosine kinase inhibitors with growth IC50 values typically greater than 1 μM, outside the clinically relevant range [6–10]. Because not all cells are sensitive to EGFR inhibitors, there is a need to identify cells sensitive to EGFR inhibition. This has been accomplished through cellular morphology using representative signaling proteins, specifically, E-cadherin and β-catenin for EGFR sensitive epithelial type cells, and vimentin for EGFR insensitive mesenchymal type cells [6,11–13].

EGFR inhibitors have been studied for their potential clinical application in PDAC, beginning in earnest after a 2007 phase III clinical trial showed increased survival in non-resectable PDAC patients treated with the EGFR inhibitor erlotinib in combination with the first-line PDAC chemotherapy gemcitabine [14]. Since the initial trial, studies have produced mixed results, with a phase III trial of the EGFR inhibitor cetuximab in combination with gemcitabine showing no improvement over gemcitabine alone, as well as a recent phase III trial in resectable pancreatic cancer also showing no additional survival benefits with the erlotinib/gemcitabine combination over gemcitabine alone [15,16].

In this study, we tested an EGFR tyrosine kinase inhibitor over a range of doses encompassing the EGFR enzyme IC50 (30 nM), EGFR cell proliferation IC50 (40 to 60 nM), and the previously reported IC50s for PDAC cells (~ 5 to 15 μM), while acknowledging that concentrations above 1 μM are unlikely to have clinical efficacy [17,18]. The reversible tyrosine kinase inhibitor gefitinib was chosen over erlotinib due to its more specific EGFR effects at the clinically relevant doses [19]. We examined gefitinib in parallel with the EGFR blocking antibody cetuximab in order to compare the relative effectiveness of each drug.

Based on previous findings, we aimed to identify cellular markers and signaling pathways paramount to EGFR inhibitor sensitivity in PDAC. In PDAC cells we compared the relative expression of the morphology representative signaling proteins, E-cadherin, β-catenin, and vimentin, and the mesothelin protein, which is overexpressed in 80% or more of PDAC [20]. In addition to the cellular morphology, we assessed autocrine signaling by screening a panel of EGF ligands, including transforming growth factor α (TGF-α), which has previously been shown to be a predictor of gefitinib sensitivity [8]. Altogether, this study sought to determine predictive factors and the cellular mechanisms underlying EGFR inhibition using a tyrosine kinase inhibitor (gefitinib) and EGFR blocking antibody (cetuximab), and if this data could be used to assess the effectiveness of combination therapy with gemcitabine or an alternative drug that may be better-suited based on the cellular profile.

## Materials and methods

### Cells, antibodies, and reagents

Human PDAC cell lines were purchased from the American Type Culture Collection (ATCC) and were authenticated by DNA fingerprinting at the University of Texas MD Anderson Characterized Cell Line Core. Cells were cultured as previously described^14^. Antibodies were purchased from Cell Signaling Technologies (Danvers, MA), Invitrogen (Carlsbad, CA), Sigma-Aldrich (St. Louis, MO), Santa Cruz Biotechnology (Dallas, TX), and Abcam (Cambridge, UK). Details of the antibodies are shown in S1 Table. The STAT-3 inhibitor was supplied by David Tweardy and was previously characterized in Bharadwaj et al. [21].

### Western blot

Western blot was performed as described previously [22]. For total cell lysates, cells were solubilized in RIPA Buffer (Sigma-Aldrich, St. Louis, MO), standardized with a BCA protein assay (ThermoFisher Scientific, Waltham, MA) and then prepared for loading with 2X Laemmli Buffer (Bio-Rad, Hercules, CA). Fractionated cells were prepared using a NE-PER Nuclear and Cytoplasmic Extraction Kit (ThermoFisher Scientific, Waltham, MA). Lysates were loaded onto a 10% SDS-PAGE gel and then transferred overnight to a nitrocellulose membrane (GE, Schenectady, NY). After blocking with 5% milk in TBS, the membrane was incubated overnight at 4°C with primary antibody (S1 Table). The following day, the membrane was incubated with anti-mouse or anti-rabbit HRP-conjugated secondary antibody (Cell Signaling Technologies, Danvers, MA), followed by chemiluminescent substrate (GE, Schenectady, NY). The membrane was exposed to X-ray film (Denville Scientific, Metuchen, NJ), and then developed with a Kodak X-GMAT 2000 (Eastman Kodak, Rochester, NY).

### Cell viability assay (MTT)

Cells were plated onto 96-well plates at densities between 2*10^3^ and 5*10^3^ cells per well. The following day, the media was removed and the indicated drugs were added in media containing 1% FBS. At the indicated times, MTT was added to the wells at a final concentration of 500 μg/ml and the cells were incubated at 37 °C for 2 hours. Afterwards, the medium was removed and the cells were solubilized by the addition of 50 μl of DMSO (ThermoFisher Scientific, Waltham, MA) per well. Absorbance was measured at 560 nm. Percent viability was measured by dividing OD value of the treated cells by that for untreated cells multiplied by 100.

### Cell viability (Annexin V, PI staining)

Cells were plated at a density of 5 x 10^4^ cells/well in 6-well plates in 2 ml of DMEM (10% FBS). The following day the media was removed, the wells were washed with PBS, and DMEM (1% FBS) was added to each well containing the indicated drugs. The cells were incubated with the indicated drugs for the indicated time period. Media was then removed and the cells were disassociated with Accutase (BD Biosciences, San José, CA) for 5 min at 37 °C. The cells were removed and centrifuged at 400 x g for 5 minutes. Cells were washed once in binding buffer (10 mM HEPES, 2.5 mM CaCl_2_, 140 mM NaCl). FITC conjugated Annexin V (BD Biosciences, San José, CA) and 1 μg/ml propidium iodide (PI; Sigma-Aldrich, St. Louis, MO) in binding buffer were added to cells and incubated at RT for 20 minutes. Analysis was done on a BD LSR II Flow Cytometer (BD Biosciences, San José, CA).

### Cell cycle analysis

Cells were plated at a density of 5 x 10^4^ cells per well in 6-well plates in 2 ml of DMEM (10% FBS). The following day the media was removed, the wells were washed with PBS, and DMEM (1% FBS) was added to each well containing the indicated drugs. The cells were incubated with the indicated drugs for 24 hours, at which point media was removed and the cells were disassociated with Accutase for 5 minutes at 37°C. Suspended cells were collected and centrifuged at 300 x g for 5 minutes. The pelleted cells were then fixed in 70% ice cold ethanol. The cells were fixed overnight at 4°C. The following morning the cells were centrifuged for 5 min at 850 × g and the supernatant was removed. The cells were washed once with PBS to remove excess ethanol and resuspended in PBS containing 20 μg/ml propidium iodide (PI), 10 μg/ml RNase A, and 0.01% Triton X-100. The cells were incubated for 30 minutes at 37°C and then analyzed on a BD LSR II Flow Cytometer.

### Real-time polymerase chain reaction (RT-PCR)

Cells were plated at a density of 1x10^6^ cells/well onto 10 cm dishes in 10 ml of DMEM (+10% FBS). The following day, cells were detached with Accutase, collected, and centrifuged at 400 x g for 5 min. RNA was extracted using Trizol Reagent (ThermoFisher Scientific, Waltham, MA) and the standard chloroform extraction protocol. Synthesis of cDNA was performed with iScript (Bio-Rad, Hercules, CA) reverse transcriptase with loading of 2 μg of RNA. RT-PCR was performed on a Bio-Rad iCycler (Bio-Rad, Hercules, CA) with SYBR Green (Bio-Rad, Hercules, CA) and the indicated primers (S2 Table).

### Statistical analysis

Data from treated and control groups were analyzed and results were presented as the arithmetic mean ± standard error mean (SEM). Statistical analysis was done with Student’s unpaired t-test, one-way ANOVA, and Tukey post-hoc test, for comparison of multiple groups, or with two-way ANOVA and the Bonferonni post-hoc test for comparison of parametric data between two or more groups. The Kruskal-Wallis test or the Mann-Whitney test was used for non-parametric data. Drug combination effects were analyzed and a combination index (CI) calculated with CompuSyn Software (ComboSyn, Inc., Paramus, NJ) [23]. Graphpad Prism was used to calculate statistics (Graphpad Software, Inc., La Jolla, CA). A value of *p* < 0.05 was considered significant.

## Results

### PDAC cell sensitivity to EGFR inhibitors

**Fig 1:**
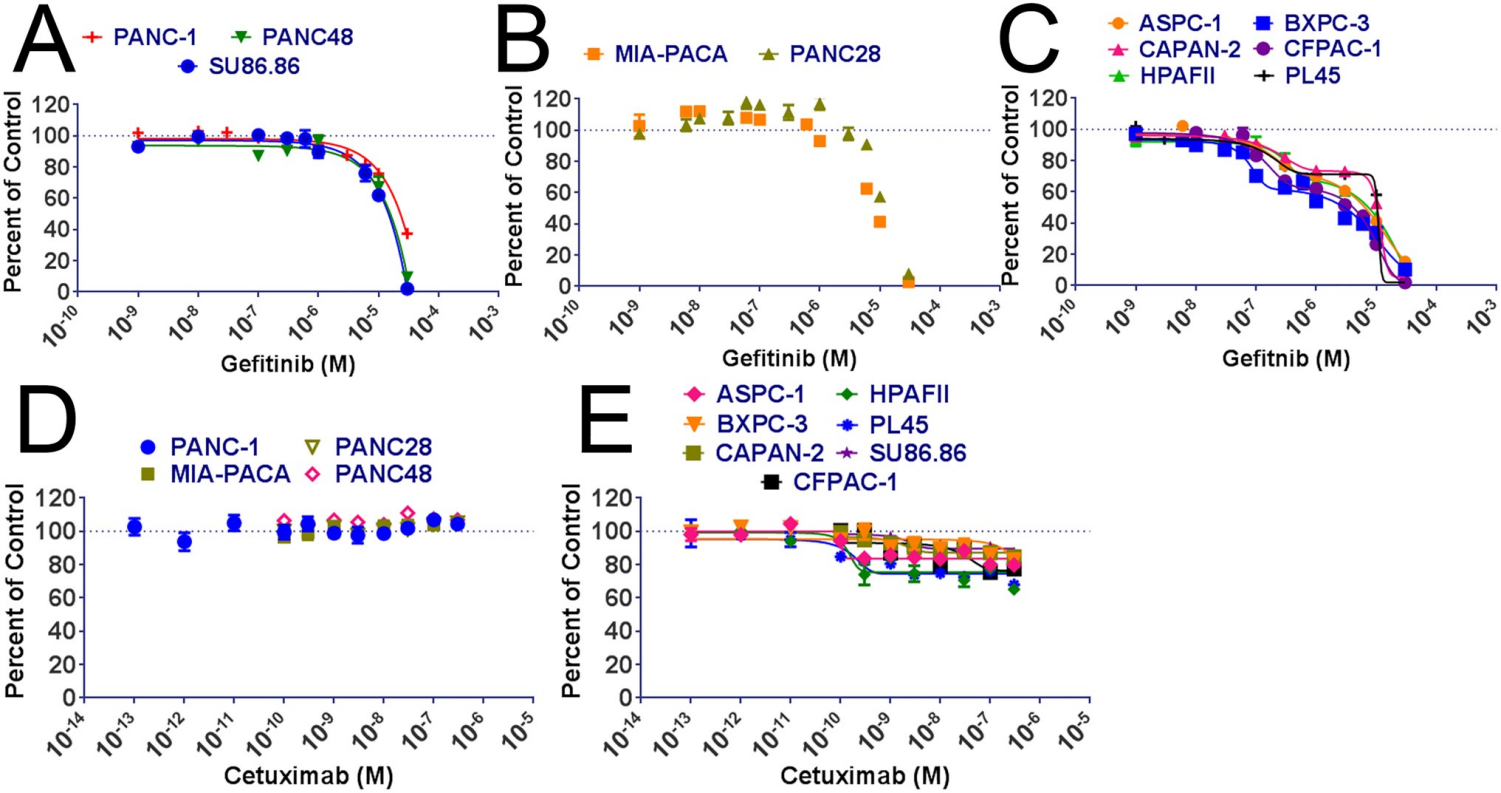
EGFR inhibitor sensitivity on selected pancreatic adenocarcinoma cell lines. PDAC cells were treated with the indicated concentrations of gefitinib for 6 days and cell viability was measured by MTT assay. (A) Gefitinib insensitive cells. Monophasic curves were generated for gefitinib insensitive MTT data. (B) Gefitinib activated cells. No curve was generated for gefitinib activated cells; (C) Gefitinib sensitive cells. A biphasic curve was generated for gefitinib sensitive MTT data. PDAC cells were treated with the indicated concentrations of cetuximab for 6 days and cell viability was measured by MTT assay. (D) Cetuximab insensitive cells. No curve was generated for cetuximab insensitive cells. (E) Cetuximab sensitive cells. Monophasic curves were generated for cetuximab sensitive MTT data. Assays were completed in triplicate.

We screened 11 PDAC cell lines for their sensitivity to the EGFR tyrosine kinase inhibitors gefitinib and cetuximab. The PDAC cells were incubated with gefitinib at concentrations ranging between 1 nM to 30 μM for 6 days and then cell viability was measured with the MTT assay. Two inhibitory responses were observed from gefitinib treatment: 1) monophasic inhibition with an average IC50 of 8.3 μM; and 2) a biphasic inhibitory response with an average IC50_1_ of 56 nM and an average IC50_2_ of 7.1 μM (Fig 1A and 1C; Table 1). Surprisingly, in contrast to the inhibitory effect, we observed increases in cell number after gefitinib treatment in the Panc-28 and MIA-Paca cell lines (Fig 1B).

**Table 1:** PDAC IC50 when treated with gefitinib or cetuximab.

	Gefitinib		Cetuximab
**Cell Line**	**IC50 1 (**μ**M)**	**IC50 2 (**μ**M)**	**IC50 (nM)**
**ASPC-1**	0.061	9.0	0.104
**BXPC3**	0.074	6.7	24.8
**CAPAN-2**	0.048	11	1.22
**CFPAC-1**	0.076	6.1	19.9
**HPAFII**	0.038	8.0	0.141
**PL45**	0.137	12.5	0.147
**Cell Line**	**IC50 1 (**μ**M)**		**IC50 (nM)**
**MIA-PACA2**	7.7		NA
**PANC-1**	11		NA
**PANC-28**	8.1		NA
**PANC-48**	15		NA
**SU86.86**	14		1.86

The same procedures were used for measuring the effects of cetuximab treatment. The 11 PDAC cells were treated with cetuximab concentrations ranging from 100 fM to 300 nM for 6 days, and then cell viability was measured by MTT. An inhibitory response of approximately 20% was observed in 7 of the cell lines between the concentrations of 100 pM and 100 nM, while the other 4 cell lines had no change in cell viability at the concentrations used (Fig 1D and 1E; Table 1). In contrast to the gefitinib treatment, we observed no increases in cell number in the MIA-Paca or Panc-28 cell lines when treated with cetuximab.

### Mesothelin expression predicts EGFR inhibitor sensitivity

**Fig 2:**
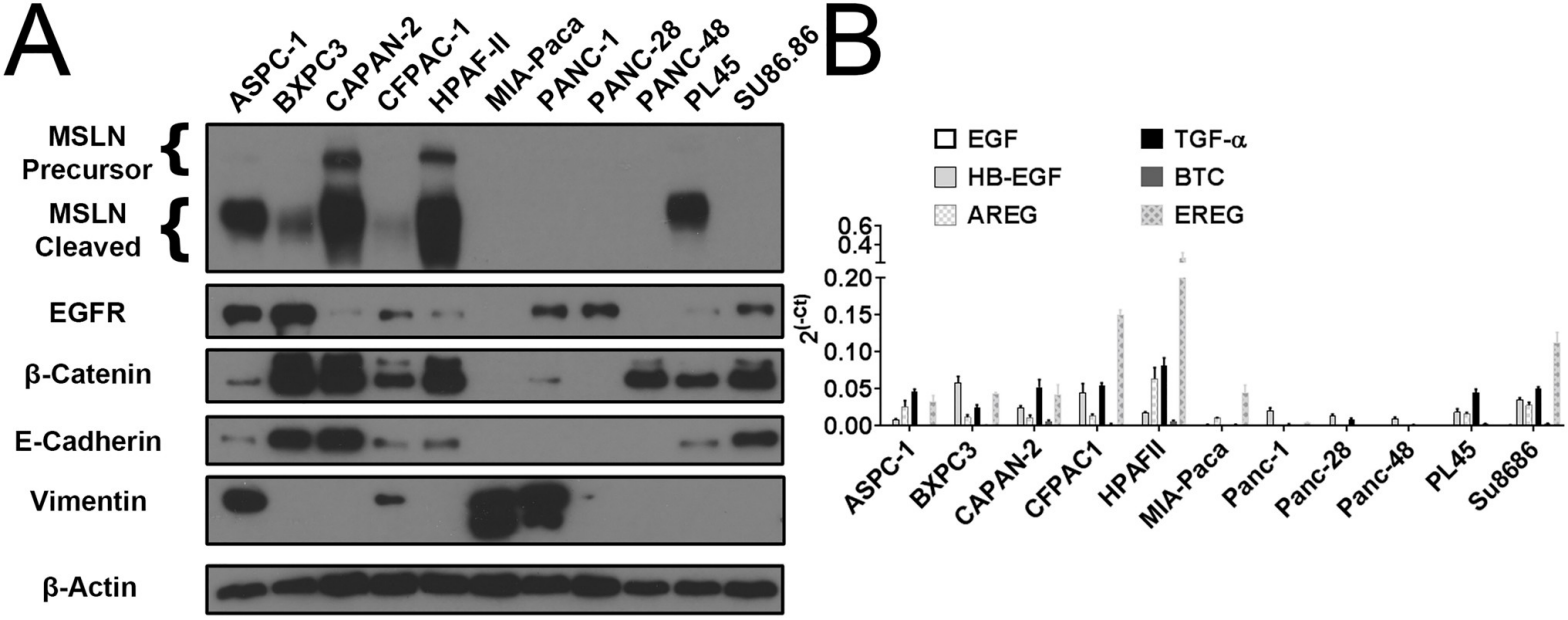
Expression levels of important cellular markers associated with EGFR sensitivity. (A) Protein expression levels of mesothelin (MSLN), EGFR, β-Catenin, E-cadherin, and vimentin in 11 PDAC cell lines detected by western blot; (B) RNA expression levels of the EGFR ligands including EGF, Heparin-binding EGF (HB-EGF), Amphiregulin (AREG), Transforming Growth Factor α (TGF-α), Betacellulin (BTC), and Epiregulin (EREG) detected by RT-PCR. Y-axis represents relative mRNA levels to GAPDH control.

We then screened the 11 PDAC cell lines to compare the relative expression levels of known markers for EGFR sensitivity: E-cadherin, vimentin, and β-Catenin, along with EGFR and mesothelin (Fig 2A). Using Boolean correlations to densitometric protein expression, we analyzed whether expression of the indicated proteins correlated to gefitinib sensitivity, defined as sensitive if the IC50_1_ value was less than 100 nM (Table 2). Of the screened proteins, only mesothelin expression was significantly correlated to gefitinib sensitivity. While a trend was observed with E-cadherin expression, it was not significant. Alternative approaches for determining correlations were considered, such as using IC50_1_ values (IC50_1_ values vs. densitometric protein expression) and visual detection (Boolean values for sensitivity vs. Boolean values for protein detection). Using IC50_1_ values yielded a similar result as using the Boolean correlation. On the other hand, visual detection on a western blot showed both mesothelin and E-cadherin to be significantly correlated to gefitinib sensitivity (S3 Table).

**Table 2:** Correlations between protein expression and EGFR inhibitors sensitivity.

**Correlation of Protein Expression and Gefitinib Sensitivity**					
	**Mesothelin**	**EGFR**	β**-Catenin**	**E-Cadherin**	**Vimentin**
R^2^ Value	0.4938	0.1126	0.1786	0.1963	0.001359
P Value	0.0159	0.3131	0.1953	0.1724	0.9143
**Correlation of Protein Expression and Cetuximab Sensitivity**					
	**Mesothelin**	**EGFR**	β**-Catenin**	**E-Cadherin**	**Vimentin**
R^2^ Value	0.5322	0.05248	0.3367	0.4222	0.09032
P Value	0.0108	0.498	0.0613	0.0305	0.3692

Using the same criteria as gefitinib, the densitometric protein expression was compared to cetuximab sensitivity using a Boolean correlation, with sensitive cells being defined by having an IC50 value below 100 nM (Table 2). We found that both mesothelin and E-cadherin expression significantly correlated to cetuximab sensitivity, and we observed a positive trend with β-Catenin expression.

We then measured the transcription levels of six major EGF ligands: EGF, heparin-binding EGF (HB-EGF), amphiregulin (AREG), transforming growth factor α (TGF-α), betacellulin (BTC), and epiregulin (EREG), by RT-PCR in the 11 PDAC cell lines (Fig 2B). For both cetuximab and gefitinib, only TGF-α transcription was significantly correlated to sensitivity to EGFR inhibition (Table 3). However, there was a trend towards correlation between inhibition and transcription levels of AREG, EREG, and BTC.

**Table 3:** Correlations between EGFR ligand transcription to EGFR inhibitors sensitivity.

**Correlation of Transcription and Gefitinib Sensitivity**						
	EGF	HB-EGF	AREG	TGF-α	BTC	EREG
R^2^ Value	0.09558	0.1465	0.213	0.5303	0.2247	0.129
P Value	0.3549	0.2453	0.153	0.011	0.1408	0.279
**Correlation of Transcription and Gefitinib Sensitivity**						
	EGF	HB-EGF	AREG	TGF-α	BTC	EREG
R^2^ Value	0.05212	0.2946	0.362	0.7695	0.3181	0.241
P Value	0.4996	0.0845	0.0502	0.0004	0.0707	0.125

### Gefitinib inhibits cell cycle progression

To determine the mechanisms by which gefitinib treatment induced a decrease the number of cells as indicated by the MTT assay, cells were analyzed for cell death with PI/annexin-V staining and cell cycle arrest by PI staining and flow cytometry for cell cycle. Cells were treated with vehicle control, 100 nM gefitinib, or 10 μM gefitinib for 24 hours and were then stained with propidium iodide and annexin-V to determine the number of healthy (PI^-^, annexin V^-^), early apoptotic (PI^-^, annexin V^+^), late apoptotic (PI^+^, annexin V^+^), and necrotic (PI^+^, annexin V^-^) cells. No significant change was observed in any group after 24 hours of gefitinib treatment, although a trend of increased percentage of healthy cells was observed in MIA-Paca cells (S1 Fig).

Cell cycle analysis was performed by synchronization via serum deprivation followed by treatment for 24 hours with vehicle control, 100 nM gefitinib, or 10 μM gefitinib. While considerable differences between cell lines were observed in the percentage of cells dividing, only minor changes and no overriding trend was observed with 100 nM gefitinib treatment (S2 Fig). On the other hand, 24 hours of 10 μM gefitinib induced cell cycle inhibition in all 6 cell lines, as indicated by an increase in G1 Phase and a decrease in S Phase and G2/M Phase. Because growth inhibition was not observed at 100 nM gefitinib, CAPAN-2, ASPC-1, and BXCP-3 cells were measured with MTT over a six day period after treatment with 100 nM and 10 μM gefitinib (S3 Fig). For both CAPAN-2 and ASPC-1 cells, 100 nM gefitinib did not significantly decrease growth compared to control until day 6, while 10 μM gefitinib showed affects by day 3.

### Gefitinib inhibits PDAC plc-γ1 and c-myc at clinical concentrations

**Fig 3:**
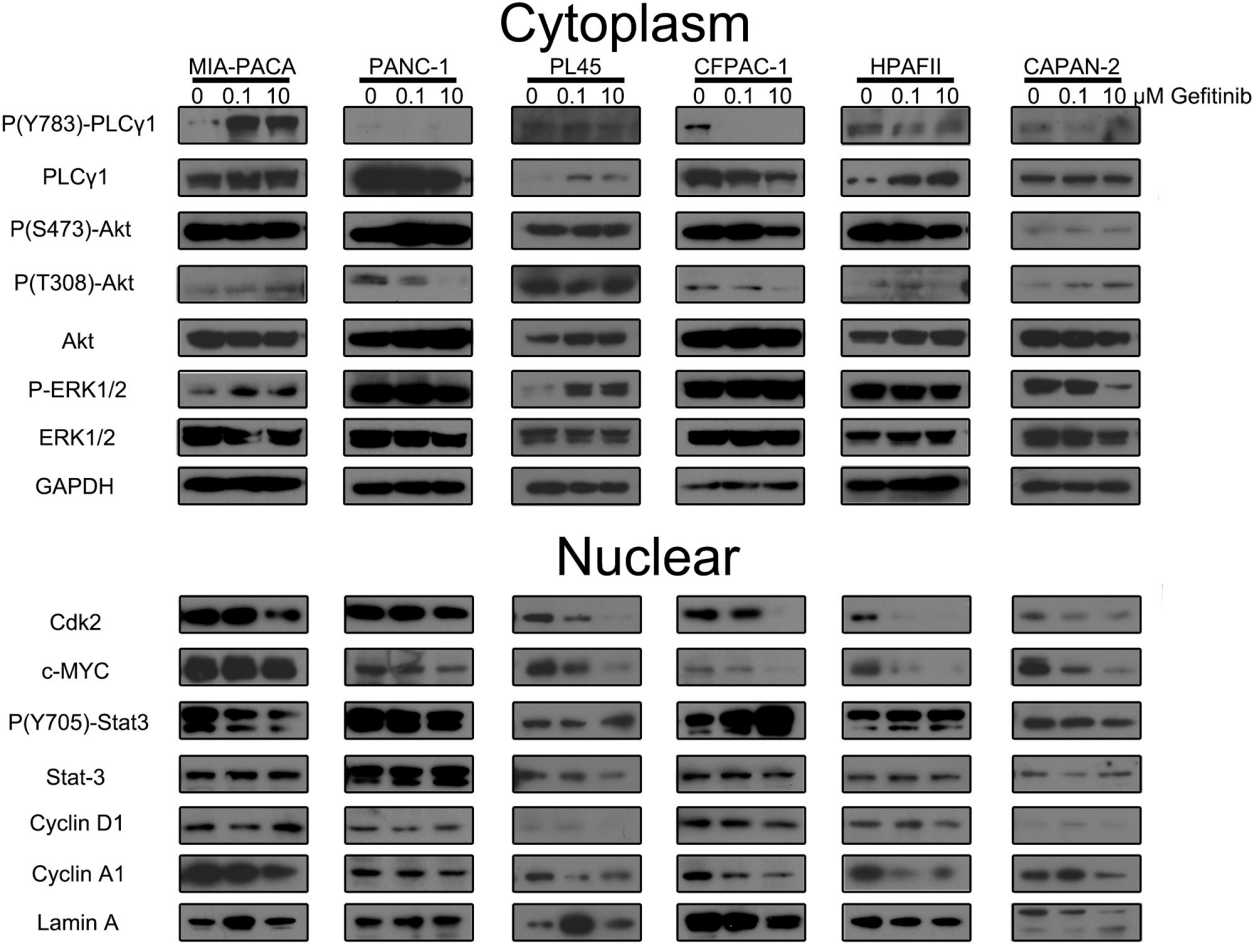
Effects of gefitinib on EGFR downstream signaling pathways. PDAC cell lines were treated with 100 nM gefitinib, 10 μM gefitinib or vehicle control for 24 hours, cells were lysed and fractionated to cytoplasmic and nuclear fractions. Western blots were used to detect total or phosphorylated protein changes in the EGFR downstream signaling pathways in the identified cellular compartments. GAPDH was used as the cytoplasmic protein internal control and Lamin A was used as the nuclear protein internal control.

Four major downstream pathways of EGFR were screened to determine how gefitinib treatment affected each pathway in cell lines chosen from our initial screen: 4 sensitive, 1 insensitive, and 1 excitatory. Three cytoplasmic pathways, ERK1/2, Akt, and plc-γ1, and one nuclear pathway, STAT3, were assessed based on their phosphorylation after gefitinib treatment. Cells were treated with vehicle control, 100 nM gefitinib, or 10 μM gefitinib for 24 hours and then fractionated into nuclear and cytoplasmic components, and analyzed by western blot (Fig 3). Of the four major pathways screened, gefitinib consistently inhibited plc-γ1 as demonstrated by decreased phosphorylation of Y783 compared to total protein in all four sensitive cell lines (CFPAC-1, HPAF-II, PL45, and CAPAN-2). Furthermore, we observed an increase in growth after gefitinib treatment in the MIA-PACA cells, which was matched by increased activation of plc-γ1 after gefitinib treatment. We did not observe any consistent inhibition or activation of the ERK, Akt, or STAT3 pathways following gefitinib treatment.

Downstream modulators of cell cycle were also affected in gefitinib sensitive cells, specifically inhibition of cyclin A1 and its associated kinase CDK2, but not cyclin D1. Furthermore, inhibition of cyclin A1 was accompanied by downregulation of nuclear c-myc in the gefitinib sensitive cell lines.

### EGFR inhibitors in combination with trametinib consistently produced additive effects

**Fig 4:**
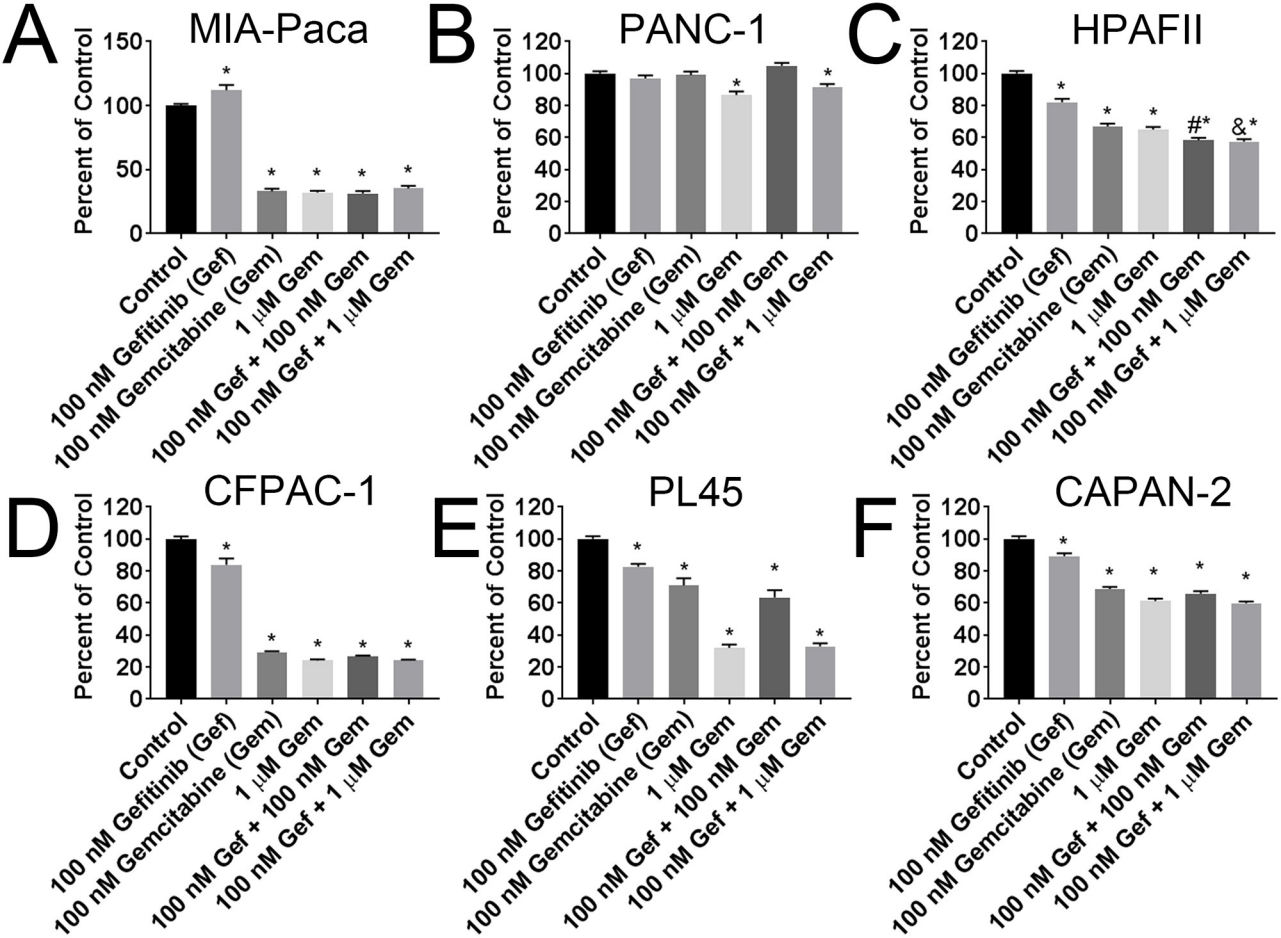
Combination treatment of gefitinib and gemcitabine in select cell lines. MTT of 3-day treatment of the 100 nM gefitinib alone or in combination with 100 nM or 1 μM gemcitabine in (A) MIA-PACA, (B) PANC-1, (C) CFPAC-1, and (D) HPAF-II. MTT of 6 day treatment of the 100 nM gefitinib alone or in combination with 100 nM or 1 μM gemcitabine in (E) PL45, and (F) CAPAN-2 cells. * denotes p <0.05 when compared to control by one-way ANOVA and Tukey post-test. # denotes p <0.05 when compared to 100 nM gefitinib alone and 100 nM gemcitabine alone by one-way ANOVA, Tukey post-test, and Chou Talalay CI value equal to or less than 1. & denotes p <0.05 when compared to 100 nM gefitinib alone and 1 μM gemcitabine alone by one-way ANOVA, Tukey post-test, and Chou Talalay CI value equal to or less than 1. Assays were completed in triplicate.

In light of the cell signaling data implicating plc-γ1 as the likely pathway of gefitinib inhibition, we combined gefitinib with inhibitors of stat3 (CMPD-1893), MEK1/2 (trametinib), mTOR (rapamycin), and compared these to the previously indicated chemotherapeutic gemcitabine to identify optimal drug combinations. In addition, we analyzed gefitinib, trametinib, and gemcitabine combination using Compusyn software to detect if the observed effects met the criteria for antagonistic (CI > 1), additive (CI = 1), or synergistic (CI < 1) effects. The PDAC cell lines were screened for combination treatment of 100 nM gefitinib with 100 nM gemcitabine or 1 μM gemcitabine (Fig 4). No significant differences were observed between gefitinib alone and gefitinib and gemcitabine co-treatment except in HPAF-II cells, where both 100 nM and 1 μM gemcitabine exhibited additive effects with 100 nM gefitinib as compared to either drug alone (Fig 4C; Table 4).

**Table 4:** Chou-Talalay CI values in EGFR inhibitors combination treatment with gemcitabine and trametinib.

**100 nM** **Gefitinib**		MIA-Paca2		PANC-1		HPAFII		CFPAC-1		PL45		CAPAN-2	
		Effect	CI Value	Effect	CI Value	Effect	CI Value	Effect	CI Value	Effect	CI Value	Effect	CI Value
Trametinib (M)	1.00E-08	0.3353	1.93	0.0313	NaN	0.4843	0.49	0.3758	0.21	0.3993	0.39	0.7608	0.36
	1.00E-07	0.5149	1.45	0.0264	NaN	0.7843	0.76	0.4749	0.12	0.4546	1.46	0.861	0.73
Gemcitabine (M)	1.00E-07	0.6858	0.03	1.00E-4	516.37	0.4165	0.05	0.7342	0.33	0.3647	0.77	0.3419	0.48
	1.00E-06	0.6468	3571.23	0.0833	1.61	0.4243	0.04	0.7596	1.00	0.6718	1.08	0.4018	0.72
**100 nM** **Cetuximab**		MIA-Paca2		PANC-1		HPAFII		CFPAC-1		PL45		CAPAN-2	
		Effect	CI Value	Effect	CI Value	Effect	CI Value	Effect	CI Value	Effect	CI Value	Effect	CI Value
Trametinib (M)	1.00E-08	0.6303	4.08E+05	0.001	NaN	0.6646	0.38	0.3688	0.53	0.405	1.00	0.4554	0.30
	1.00E-07	0.7562	9.20E+05	0.001	NaN	0.7851	0.72	0.3805	0.69	0.4895	0.64	0.4582	2.72
Gemcitabine (M)	1.00E-07	0.7666	9.94E+05	0.3817	1.30E+10	0.4903	0.42	0.8005	0.72	0.4524	0.80	0.2772	0.73
	1.00E-06	0.7674	1.00E+06	0.7011	4.06E+12	0.5956	0.13	0.8508	1.16	0.6377	0.75	0.5053	1.16

**Fig 5:**
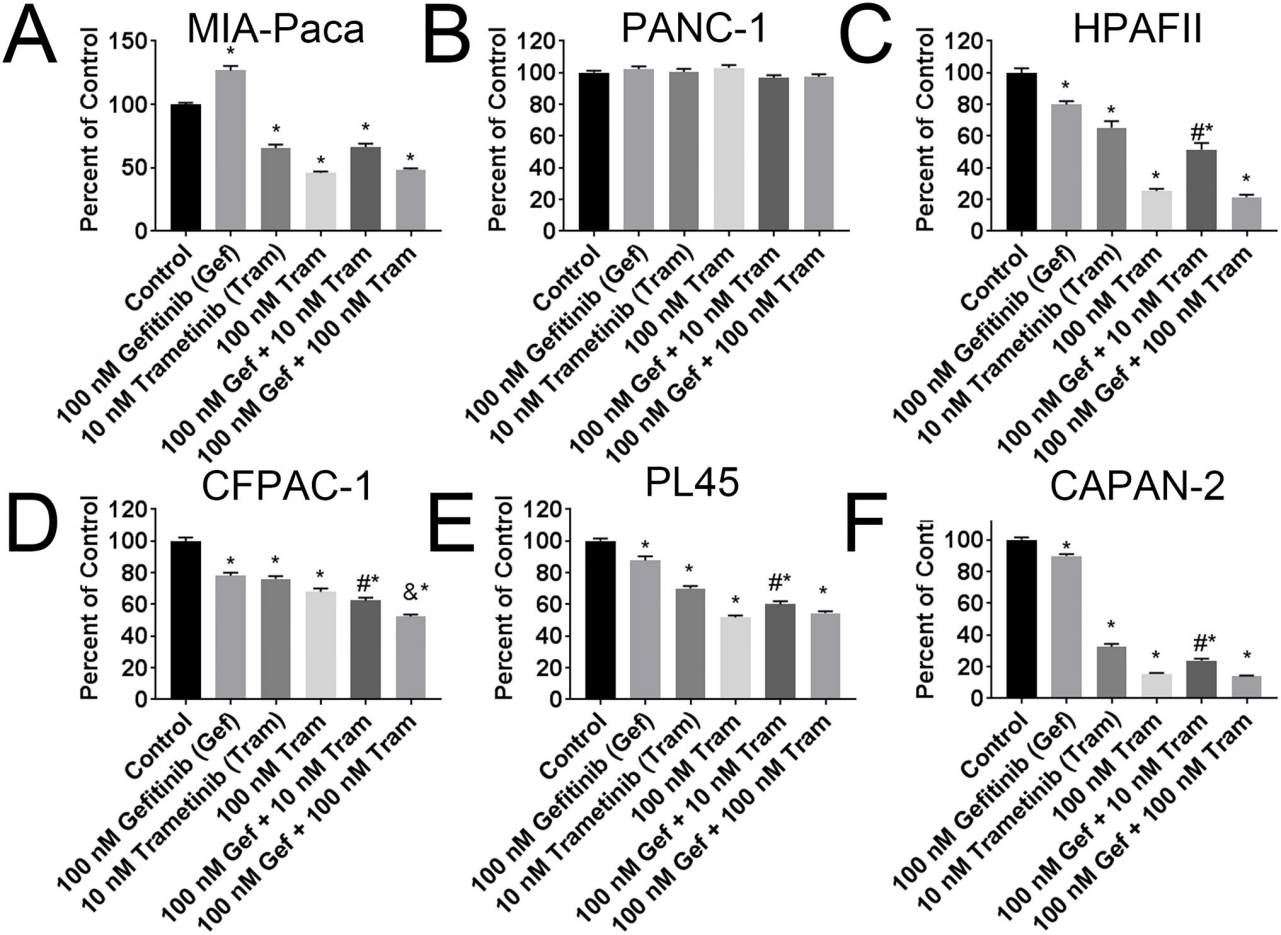
Combination treatment of gefitinib and trametinib in select cell lines. MTT of 3-day treatment of the 100 nM gefitinib alone or in combination with 10 nM or 100 nM trametinib in (A) MIA-PACA, (B) PANC-1, (C) CFPAC-1, and (D) HPAF-II. MTT of 6 day treatment of the 100 nM gefitinib alone or in combination with 10 nM or 100 nM trametinib (E) PL45, and (F) CAPAN-2 cells. * denotes p <0.05 when compared to control by one-way ANOVA and Tukey post-test. # denotes p <0.05 when compared to 100 nM gefitinib alone and 10 nM trametinib alone by one-way ANOVA, Tukey post-test, and Chou Talalay CI value equal to or less than 1. & denotes p <0.05 when compared to 100 nM gefitinib alone and 100 nM trametinib alone by one-way ANOVA, Tukey post-test, and Chou Talalay CI value equal to or less than 1. Assays were completed in triplicate.

Next, the ERK1/2 pathway inhibitor, trametinib was used in combination with gefitinib. Cells were treated with 10 nM or 100 nM trametinib alone or in combination with 100 nM gefitinib. The concentration of 10 nM trametinib consistently produced significant differences between gefitinib and trametinib alone compared to combination gefitinib and trametinib in all four sensitive cell lines (Fig 5C, 5D, 5E, and 5F; Table 4). The downstream complete inhibition of p-ERK in the combination therapy is further confirmed (S4 Fig). No additive effect was observed in the gefitinib insensitive or excitatory cell lines (Fig 5A and 5B).

The 6 cell lines were screened with the STAT3 inhibitor CMPD-1893 at concentrations of 100 nM and 1 μM, in combination with 100 nM gefitinib. Combination gefitinib and CMPD-1893 produced significant differences in one sensitive cell line, CFPAC-1, but not in PL45, CAPAN-2, or HPAF-II cells (S5 Fig). Finally, the 6 cell lines were also screened with the mTOR inhibitor rapamycin at concentrations of 10 nM and 100 nM, in combination with 100 nM gefitinib. Combination gefitinib and rapamycin produced significant differences compared to either drug alone in two sensitive cell lines, PL45 and CFPAC-1, but not in CAPAN-2 or HPAF-II cells (S6 Fig).

To determine whether the observed combination effects of gefitinib could be applied to other EGFR inhibitors, 100 nM cetuximab was combined with either gemcitabine or trametinib using a similar assay design. The previously indicated 6 PDAC cell lines were treated with 100 nM or 1 μM gemcitabine alone, or in combination with 100 nM cetuximab (S7 Fig). Only PL45 showed a significant difference to the two drugs alone with combination 100 nM cetuximab and 100 nM gemcitabine (S7E Fig). Because trametinib had shown the most comprehensive effects in combination with gefitinib, we measured the effects of combination cetuximab with 10 nM or 100 nM trametinib. Similar to the gefitinib treatment, combination cetuximab and trametinib produced significant effects in HPAF-II, CFPAC-1, PL45, and CAPAN-2 cells (S8 Fig), but not in the cetuximab insensitive cells, MIA-Paca and Panc-1.

## Discussion

The aim of this study was to understand how EGFR inhibitors affect PDAC cells, and to determine if predictive biomarkers from other cancers could be adapted to PDAC. Furthermore, because of uncertainty regarding the clinical effectiveness of combination gemcitabine and erlotinib, we considered the cellular mechanisms underlying EGFR inhibition in PDAC, and how they could be used to select a more potent drug combination.

By way of evaluating how EGFR inhibitors affected PDAC cells, we sought to discern between specific EGFR inhibition and off-target effects by selecting the proper concentrations of gefitinib. Tyrosine kinase inhibitors can bind many of the tyrosine kinase domains of the growth factor receptors expressed on the cell, in addition to their specific kinase. In the case of gefitinib, 1–3 μM gefitinib can block the activity of fibroblast growth factor (FGF) and vascular endothelial growth factor (VEGF), implying inhibition of the FGF receptor and VEGF receptor, respectively [10]. For the EGFR, gefitinib halts cell growth at concentrations of approximately 40 to 60 nM in non-small cell lung cancer and epidermoid carcinoma cells, among others [17,24]. Moreover, clinically relevant concentrations (250 mg, daily oral administration) are reported to be between 500 nM and 1 μM, indicating gefitinib concentrations above 1 μM are non-specific drug interactions and unlikely to be clinically significant [10]. In line with the gefitinib growth IC50 values reported for other cancers, we observed biphasic growth inhibition in 6 of the 11 PDAC cell lines, with initial growth inhibition occurring around the reported gefitinib IC50 of 60 to 80 nM, and an additional growth inhibition occurring around 10 μM, likely an off-target effect. Based on the biphasic inhibition, we selected two representative doses to be used for additional analysis; the gefitinib concentration of 100 nM, which is both clinically relevant and EGFR specific, and the concentration of 10 μM, which has inhibitory effects on a range of different enzymes [19]. As a monoclonal antibody, cetuximab has much less off-target binding and has been reported to have an IC50 of approximately 200 pM to 10 nM, and upwards to 6–7 μM in some cell lines [25–27]. We observed a trend of high picomolar and low nanomolar partial growth inhibition, with IC50’s falling between 100 pM to 25 nM in this study. For combination thereapy, 100 nM cetuximab was chosen since it corresponds with concentrations used frequently in studies (100 nM is ~ 15 μg/ml) and there is a lower likelihood of off-target effects since it is a monoclonal antibody [28,29]. Both cetuximab and gefitinib at their respective clinically relevant concentrations achieved approximately 15 to 20% growth inhibition in the sensitive cell lines.

After identifying different cell lines with EGFR sensitivity, we next looked at potential morphology markers and EGF ligand autocrine signaling. E-cadherin has repeatedly been shown to be both a marker and driver of EGFR sensitivity in non-small cell lung cancer [12,13,30]. In pancreatic cancer, resistance to chemotherapy, including erlotinib, has previously been shown to be at least partially imparted by downregulation of E-cadherin [6,11]. Here, we showed a significant correlation to cetuximab sensitivity and E-cadherin expression, and depending on the correlation analysis, either a positive trend or a significant correlation between gefitinib sensitivity and E-cadherin expression. Our study confirmed findings in previous reports that TGF-α is correlated to gefitinib sensitivity in pancreatic cancer [8]. Furthermore, we confirmed that cetuximab sensitivity and TGF-α transcription levels are also correlated in a similar manner as gefitinib. Screening additional EGF ligands revealed that AREG and also BTC show positive trends in predicting EGFR sensitivity. Finally, our results indicate that mesothelin expression has a strong and consistent correlation to both gefitinib and cetuximab sensitivity. Mesothelin is over-expressed in 80–90% of PDACs. Additionally, mesothelin’s primary membrane interaction protein, MUC16, has previously been shown to activate EGFR and lead to additional EGFR expression and stabilization [31,32]. If this correlation is consistent in patient tissue, then most PDAC tumors will be sensitive to EGFR inhibitors, suggesting that the search for EGFR inhibitor combinations is more important than ever [20,33].

The results from our cell pathway analysis indicated that plc-γ1 inhibition was a consistent factor in gefitinib inhibition of EGFR. In contrast, the three other pathways (Akt, STAT3, and ERK1/2) did not exhibit any consistent effects relative to gefitinib treatment. Our study contained an important distinction from many previous pancreatic cancer studies examining EGFR inhibition in that we did not add exogenous EGF [7,34]. As our EGFR ligand screen indicated, most of the tumor cells were producing sufficient EGFR ligands to act as autocrine signaling and additional exogenous ligands might interfere with our observations. Without exogenous EGF, our results did not show ERK1/2 pathway inhibition by gefitinib treatment except in CAPAN-2 cells with high dose (10 μM) gefitinib. An important caveat is that our results do not preclude inhibition of EGFR translocation to the nucleus. Nuclear EGFR has previously been indicated to directly interact with c-myc and induce cell cycle arrest, and is a possible mechanism of the growth inhibitory effects we observed [35]. Calcium release, via activation of plc-γ1 and subsequent downstream pathways, has also been shown to induce c-myc expression [36,37]. Similarly, decreases in nuclear cyclin A1 and CDK2 leave both EGFR translocation and calcium signaling via plc-γ1 activation as possible upstream events.

Sensitivity to EGFR inhibitors is highly dependent on the specific driver mutation of either KRAS or EGFR [38]. Typically, driver mutations of these two genes are mutually exclusive, in that a cancer with an EGFR driver mutation will not harbor a KRAS driver mutation and vice versa. EGFR inhibitors are less effective when the driver mutations are not specific to EGFR and instead harbored in KRAS, which is particularly important in pancreatic cancer because KRAS is mutated in approximately 95% of PDAC [39]. Therefore, optimal growth inhibition via EGFR cannot occur in PDAC; however, combination therapy with EGFR inhibitors can still provide effective therapy when properly selected. Our combination screening of an EGFR inhibitor (gefitinib or cetuximab) and gemcitabine produced mixed results showing questionable effectiveness of either drug combination, a finding similar to that from the clinical trials. This is possibly due to opposing mechanisms of action, where gefitinib treatment induced inhibition of c-myc and cell cycle arrest, and gemcitabine’s mechanism of action is based on being incorporated into DNA, which occurs much less frequently when a cell is not actively dividing [40]. It has also been reported that erlotinib synergizes with gemcitabine by inhibiting gemcitabine’s upregulation of ERK [9]. Our study does not preclude these results, which could possibly explain the effects we observed in HPAF-II and PL45 cells. Importantly, we did not see major additive effects in the majority of cell lines tested. We therefore utilized our cell signaling data to predict via nuclear STAT3 and Akt S473 (mTor activation of Akt) that a specific inhibitor of either of these pathways might be effective in combination with an EGFR inhibitor. Furthermore, rapamycin has previously been reported to exhibit additive effects when combined with EGFR inhibitors [41]. While neither the Stat3 or mTor inhibitor showed additive effects with gefitinib in all 4 sensitive cell lines undergoing screening, rapamycin showed significant additive effects in 2 cell lines, and had some positive trends in the remaining 2, indicating potential for combined use.

Because ERK lies downstream of KRAS, EGFR cannot induce permanent inhibition in pancreatic cancer, but MEK1/2 inhibitors, which target downstream of KRAS and upstream of ERK, have been approved by the FDA for BRAF mutated melanoma and are being tested in many other cancers. In addition, a previous study showed efficacy with combination erlotinib and the MEK inhibitor RDEA119 (Refametinib) in the BXPC-3 pancreatic cancer cell line [42]. While our study deemphasized BXPC-3 cells due to their wild-type KRAS, the effectiveness of EGFR inhibitors with MEK inhibitors was inspiring [43]. For our study, all cell lines exposed to combination EGFR inhibitor treatments harbored oncogenic KRAS mutations. The EGFR MEK1/2 inhibitor combination is further supported by findings in treatment refractory PDAC patients who subsequently received combination erlotinib and selumetinib, a MEK1/2 inhibitor that recently received an orphan drug designation for neurofibromatosis type 1 [44]. In this phase II trial, only 2 of the 46 patients treated with selumetinib plus erlotinib withdrew from the trial due to toxicity [44]. Furthermore, as stated previously, gefitinib has fewer off-target effects at its therapeutic concentrations compared to erlotinib so we would expect similar or less toxicity from drug combinations of gefitinib and trametinib [19]. Finally, post-study analysis by Ko et al. found that patients with high E-cadherin responded better to EGFR and MEK inhibitor combination therapy suggesting the relevance of our identified biomarkers and supported by our findings with both gefitinib and cetuximab, which showed positive additive effects with trametinib in all 4 EGFR inhibitor sensitive cell lines (and no combination effect in the 2 EGFR inhibitor insensitive cell lines). The concentrations for trametinib and gemcitabine were based on clinically relevant concentrations slightly adjusted lower to better observe potential combination effects with the benefit of lower toxicity (Cmax trametinib = 13 μM, Cmax gemcitabine = 89.3 μM) [45].

Our study confirms previous reports that epithelial morphology is indicative of EGFR inhibitor sensitivity. Furthermore, we demonstrated that most cell lines sensitive to cetuximab were also sensitive to gefitinib, but we did not identify the underlying mechanism of why SU86.86 cells only responded to cetuximab. Without any exogenous EGF ligands added, we showed that inhibition of the EGFR consistently inactivated plc-γ1 and downregulated c-myc expression. Finally, our combination studies revealed that trametinib consistently produced additive effects when combined with gefitinib and cetuximab in all sensitive cell lines. The effectiveness of the combination EGFR inhibitors and trametinib warrants additional studies evaluating the safety and efficacy *in vivo*.

In summary, our study provides several important findings and indications for better clinical therapy choices. We demonstrates that much lower drug concentrations can be used to obtain the actual EGFR inhibition in pancreatic cancer. Furthermore, our results indicate the reason of why previous clinical trial using combination of EGFR inhibitor and gemcitabine was unsuccessful as the mechanism of action of the EGFR inhibitors (cell cycle inhibition) is contradictory to the mechanism of gemcitabine (cell death based on cell division). Additionally, this is the first study to demonstrate the effectiveness of cetuximab in PDAC cell lines and its potential use in combination therapies with MEK inhibitors. Because KRAS is a frequently mutated oncogene in PDAC, our study also demonstrates that EGFR inhibition in combination with KRAS inhibitors could potentially be a viable treatment, further supported by the clinical data from Ko et al. in Clinical Cancer Research [44].

## Supporting information

S1 Fig Gefitinib did not affect cell death in PDAC cells. PI and Annexin V staining of (A) MIA-Paca, (B) Panc-1, HPAF-II, (D) CFPAC-1, (E) PL45, and (F) CAPAN-2 cells after 24 hours of gefitinib treatment. * denotes *p* <0.05 when compared to control by two-way ANOVA and Tukey post-test. Assays were completed in triplicate.
(DOCX)

S2 Fig Gefitinib inhibition of cell cycle. Propidium iodide staining of fixed cells after 24 hours of treatment with vehicle control, 100 nM, or 10 μM gefitinib. (A) MIA-PACA, (B) PANC-1, (C) CFPAC-1, (D) HPAF-II, (E) PL45, and (F) CAPAN-2 cells. Assays were completed in triplicate.
(DOCX)

S3 Fig Time series of gefitinib inhibition of cell growth. MTT of (A) ASPC-1, (B) CAPAN-2, and (C) BXPC-3 cells after 1, 3, or 6 days of 100 nM or 10 μM gefitinib treatment. * denotes p <0.05 when compared to control by two-way ANOVA and Tukey post-test. Assays were completed in triplicate.
(DOCX)

S4 Fig ERK inhibition in combination treatment of gefitinib and trametinib. CAPAN-2, MIA-PACA, PANC-1, and PL45 cells were treated with 100 nM of gefitinib or 10 nM of trametinib or combination of gefitinib and trametinib or no treatment control for 24 h, western blot were performed on cell lysates to determine total ERK (P-42/44) and p-ERK (p-P42/44). β-action was used as loading control.
(DOCX)

S5 Fig Combination treatment of gefitinib and the Stat3 inhibitor CMPD 188–9 (CMPD) in select cell lines. MTT of 3-day treatment of the 100 nM gefitinib (Gef) alone or in combination with 100 nM or 1 μM CMPD in (A) MIA-PACA, (B) PANC-1, (C) CFPAC-1, and (D) HPAF-II. MTT of 6-day treatment with 100 nM gefitinib (Gef) alone or in combination with 100 nM or 1 μM CMPD in (E) PL45, and (F) CAPAN-2 cells. * denotes *p* <0.05 when compared to control by one-way ANOVA and Tukey post-test. # denotes p <0.05 when compared to 100 nM gefitinib alone and 100 nM CMPD alone by one-way ANOVA and Tukey post-test. & denotes p <0.05 when compared to 100 nM gefitinib alone and 1 μM CMPD alone by one-way ANOVA and Tukey post-test. Assays were completed in triplicate.
(DOCX)

S6 Fig Combination treatment of gefitinib and rapamycin in select cell lines. MTT of 3 day treatment of the 100 nM gefitinib alone or in combination with 10 nM or 100 nM rapamycin in (A) MIA-PACA, (B) PANC-1, (C) CFPAC-1, and (D) HPAF-II. MTT of 6 day treatment of the 100 nM gefitinib alone or in combination with 10 nM or 100 nM rapamycin in (E) PL45 and (F) CAPAN-2 cells. * denotes p <0.05 when compared to control by one-way ANOVA and Tukey post-test. # denotes p <0.05 when compared to 100 nM gefitinib alone and 10 nM rapamycin alone by one-way ANOVA and Tukey post-test. & denotes p <0.05 when compared to 100 nM gefitinib alone and 100 nM rapamycin alone by one-way ANOVA and Tukey post-test. Assays were completed in triplicate.
(DOCX)

S7 Fig Combination treatment of cetuximab and gemcitabine in select cell lines. MTT of 6-day treatment of the 100 nM cetuximab alone or in combination with 100 nM or 1 μM gemcitabine in (A) MIA-PACA, (B) PANC-1, (C) CFPAC-1, (D) HPAF-II, (E) PL45, and (F) CAPAN-2 cells. * denotes p <0.05 when compared to control by one-way ANOVA and Tukey post-test. # denotes p <0.05 when compared to 100 nM cetuximab alone and 100 nM gemcitabine alone by one-way ANOVA, Tukey post-test, and Chou Talalay CI values equal to or less than 1. & denotes *p* <0.05 when compared to 100 nM cetuximab alone and 1 μM gemcitabine alone by one-way ANOVA, Tukey post-test, and Chou Talalay CI values equal to or less than 1. Assays were completed in triplicate.
(DOCX)

S8 Fig Combination treatment of cetuximab and trametinib in select cell lines. MTT of 6-day treatment of the 100 nM cetuximab alone or in combination with 10 nM or 100 nM trametinib in (A) MIA-PACA, (B) PANC-1, (C) CFPAC-1, (D) HPAF-II, (E) PL45, and (F) CAPAN-2 cells. * denotes p <0.05 when compared to control by one-way ANOVA and Tukey post-test. # denotes p <0.05 when compared to 100 nM cetuximab alone and 10 nM trametinib alone by one-way ANOVA, Tukey post-test, and Chou Talalay CI values equal to or less than 1. & denotes *p* <0.05 when compared to 100 nM cetuximab alone and 100 nM trametinib alone by one-way ANOVA, Tukey post-test, and Chou Talalay CI values equal to or less than 1. Assays were completed in triplicate.
(DOCX)

S1 Table List of antibodies used in this study. (DOCX)

S2 Table List of primers used for RT-PCR. (DOCX)

S3 Table Correlation of gefitinib sensitivity to the indicated proteins. (DOCX)
