# Supplementary material for: Mesothelin and TGF-α predict pancreatic cancer cell sensitivity to EGFR inhibitors and effective combination treatment with trametinib
Source: PLoS One. 2019 Mar 28;14(3):e0213294. doi: 10.1371/journal.pone.0213294 (PMC6438513; doi:10.1371/journal.pone.0213294)

**S1 Fig**: Gefitinib did not affect cell death in PDAC cells. PI and Annexin V staining of (A) MIA-Paca, (B) Panc-1, HPAF-II, (D) CFPAC-1, (E) PL45, and (F) CAPAN-2 cells after 24 hours of gefitinib treatment. * denotes *p* <0.05 when compared to control by two-way ANOVA and Tukey post-test. Assays were completed in triplicate.


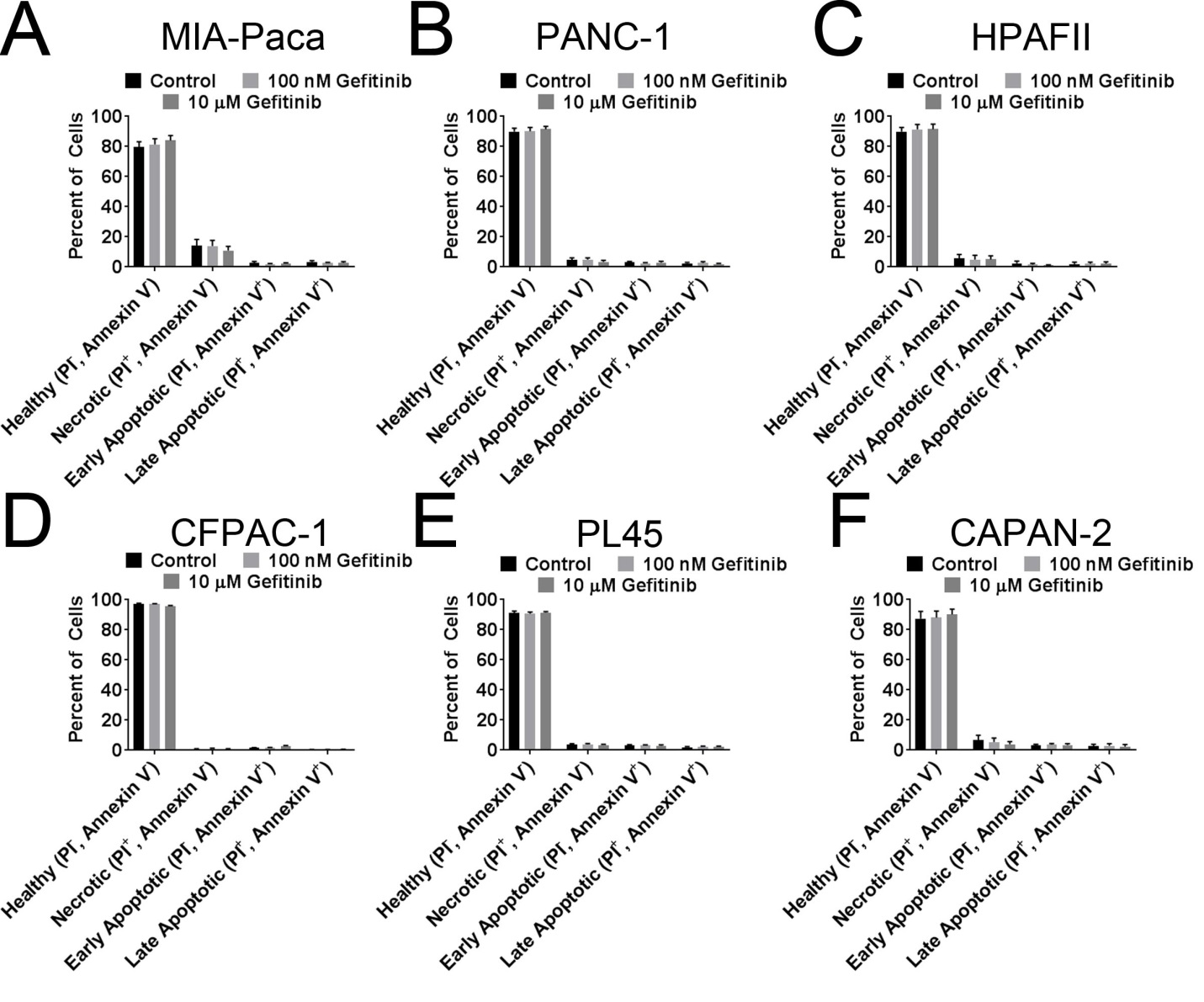

Supplement: S1 Fig — PI and Annexin V staining of (A) MIA-Paca, (B) Panc-1, HPAF-II, (D) CFPAC-1, (E) PL45, and (F) CAPAN-2 cells after 24 hours of gefitinib treatment. * denotes p <0.05 when compared to control by two-way ANOVA and Tukey post-test. Assays were completed in triplicate. (DOCX) [file pone.0213294.s001.docx]
