# Supplementary material for: Mesothelin and TGF-α predict pancreatic cancer cell sensitivity to EGFR inhibitors and effective combination treatment with trametinib
Source: PLoS One. 2019 Mar 28;14(3):e0213294. doi: 10.1371/journal.pone.0213294 (PMC6438513; doi:10.1371/journal.pone.0213294)

**S2 Fig**: Gefitinib inhibition of cell cycle. Propidium iodide staining of fixed cells after 24 hours of treatment with vehicle control, 100 nM, or 10 µM gefitinib. (A) MIA-PACA, (B) PANC-1, (C) CFPAC-1, (D) HPAF-II, (E) PL45, and (F) CAPAN-2 cells. Assays were completed in triplicate.


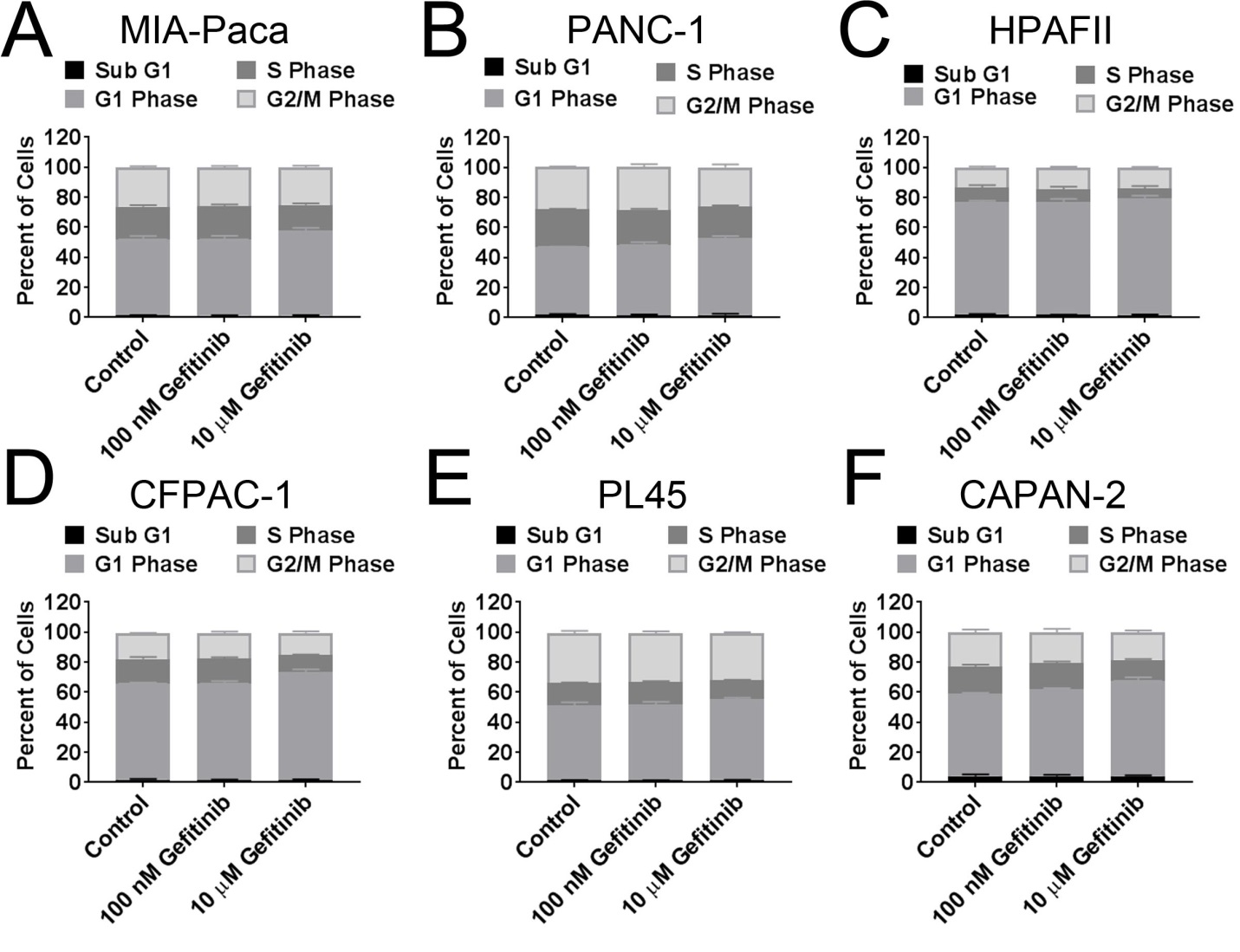

Supplement: S2 Fig — Propidium iodide staining of fixed cells after 24 hours of treatment with vehicle control, 100 nM, or 10 μM gefitinib. (A) MIA-PACA, (B) PANC-1, (C) CFPAC-1, (D) HPAF-II, (E) PL45, and (F) CAPAN-2 cells. Assays were completed in triplicate. (DOCX) [file pone.0213294.s002.docx]
