# Supplementary material for: Mesothelin and TGF-α predict pancreatic cancer cell sensitivity to EGFR inhibitors and effective combination treatment with trametinib
Source: PLoS One. 2019 Mar 28;14(3):e0213294. doi: 10.1371/journal.pone.0213294 (PMC6438513; doi:10.1371/journal.pone.0213294)

**S3 Fig:** Time series of gefitinib inhibition of cell growth. MTT of (A) ASPC-1, (B) CAPAN-2, and (C) BXPC-3 cells after 1, 3, or 6 days of 100 nM or 10 µM gefitinib treatment. * denotes p <0.05 when compared to control by two-way ANOVA and Tukey post-test. Assays were completed in triplicate.


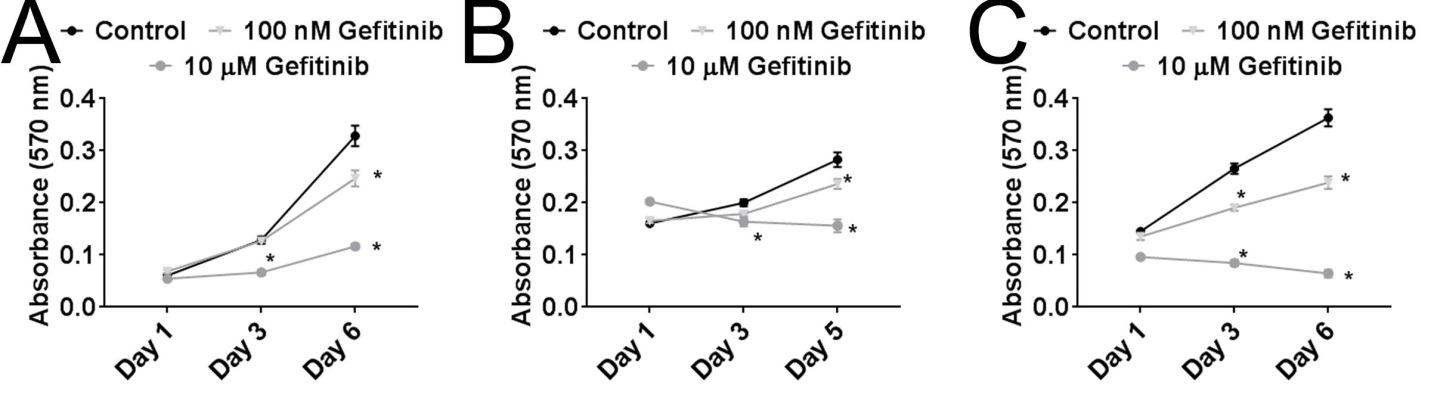

Supplement: S3 Fig — MTT of (A) ASPC-1, (B) CAPAN-2, and (C) BXPC-3 cells after 1, 3, or 6 days of 100 nM or 10 μM gefitinib treatment. * denotes p <0.05 when compared to control by two-way ANOVA and Tukey post-test. Assays were completed in triplicate. (DOCX) [file pone.0213294.s003.docx]
