# Supplementary material for: Mesothelin and TGF-α predict pancreatic cancer cell sensitivity to EGFR inhibitors and effective combination treatment with trametinib
Source: PLoS One. 2019 Mar 28;14(3):e0213294. doi: 10.1371/journal.pone.0213294 (PMC6438513; doi:10.1371/journal.pone.0213294)
Supplement: S4 Fig — CAPAN-2, MIA-PACA, PANC-1, and PL45 cells were treated with 100 nM of gefitinib or 10 nM of trametinib or combination of gefitinib and trametinib or no treatment control for 24 h, western blot were performed on cell lysates to determine total ERK (P-42/44) and p-ERK (p-P42/44). β-action was used as loading control. (DOCX) [file pone.0213294.s004.docx]

**S4 Fig:** ERK inhibition in combination treatment of gefitinib and trametinib. CAPAN-2, MIA-PACA, PANC-1, and PL45 cells were treated with 100 nM of gefitinib or 10 nM of trametinib or combination of gefitinib and trametinib or no treatment control for 24 h, western blot were performed on cell lysates to determine total ERK (P-42/44) and p-ERK (p-P42/44). β-action was used as loading control.
