# Supplementary material for: Mesothelin and TGF-α predict pancreatic cancer cell sensitivity to EGFR inhibitors and effective combination treatment with trametinib
Source: PLoS One. 2019 Mar 28;14(3):e0213294. doi: 10.1371/journal.pone.0213294 (PMC6438513; doi:10.1371/journal.pone.0213294)

**S6 Fig:** Combination treatment of gefitinib and rapamycin in select cell lines. MTT of 3 day treatment of the 100 nM gefitinib alone or in combination with 10 nM or 100 nM rapamycin in (A) MIA-PACA, (B) PANC-1, (C) CFPAC-1, and (D) HPAF-II. MTT of 6 day treatment of the 100 nM gefitinib alone or in combination with 10 nM or 100 nM rapamycin in (E) PL45 and (F) CAPAN-2 cells. * denotes p <0.05 when compared to control by one-way ANOVA and Tukey post-test. # denotes p <0.05 when compared to 100 nM gefitinib alone and 10 nM rapamycin alone by one-way ANOVA and Tukey post-test. & denotes p <0.05 when compared to 100 nM gefitinib alone and 100 nM rapamycin alone by one-way ANOVA and Tukey post-test. Assays were completed in triplicate.


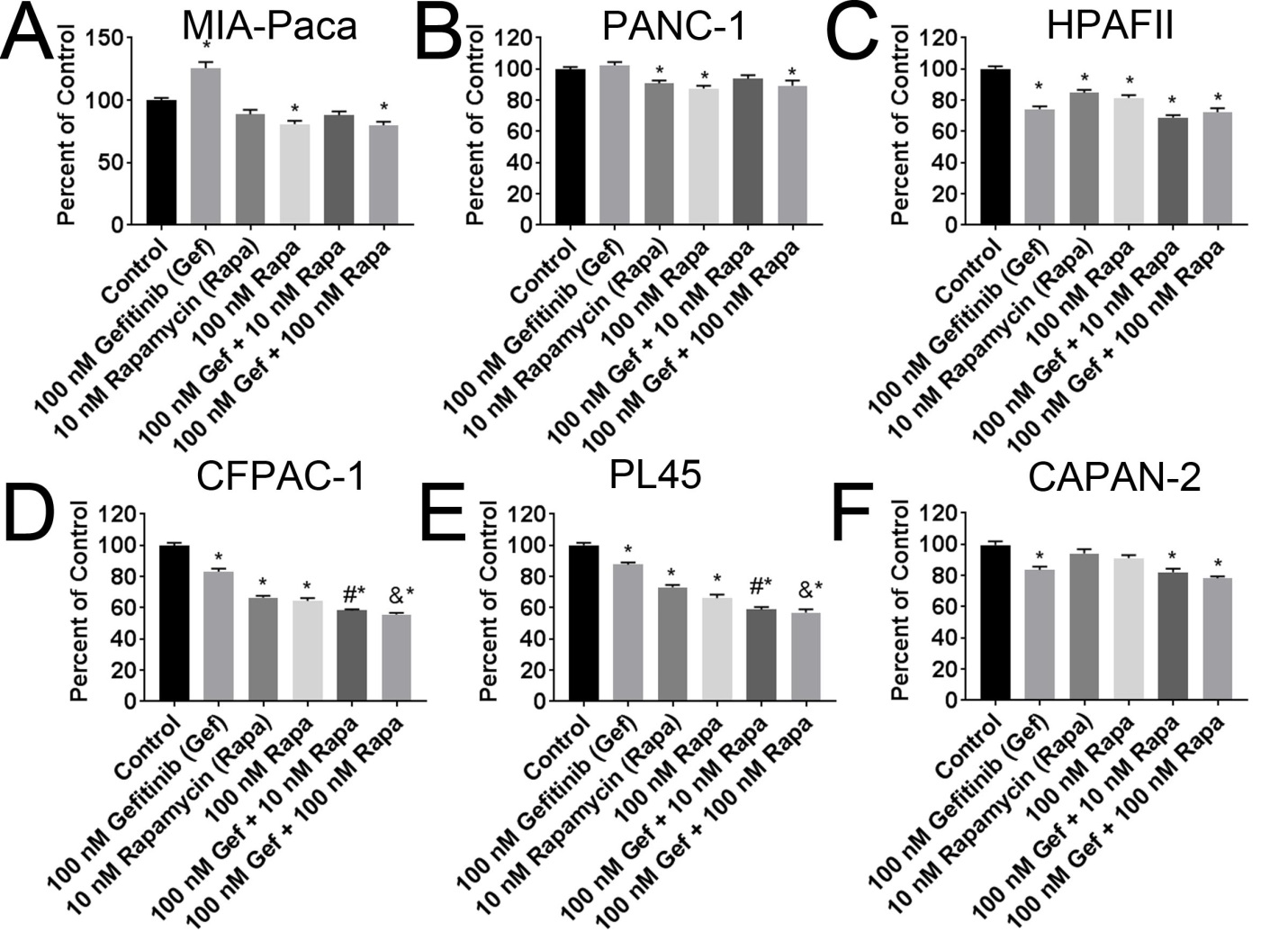

Supplement: S6 Fig — MTT of 3 day treatment of the 100 nM gefitinib alone or in combination with 10 nM or 100 nM rapamycin in (A) MIA-PACA, (B) PANC-1, (C) CFPAC-1, and (D) HPAF-II. MTT of 6 day treatment of the 100 nM gefitinib alone or in combination with 10 nM or 100 nM rapamycin in (E) PL45 and (F) CAPAN-2 cells. * denotes p <0.05 when compared to control by one-way ANOVA and Tukey post-test. # denotes p <0.05 when compared to 100 nM gefitinib alone and 10 nM rapamycin alone by one-way ANOVA and Tukey post-test. & denotes p <0.05 when compared to 100 nM gefitinib alone and 100 nM rapamycin alone by one-way ANOVA and Tukey post-test. Assays were completed in triplicate. (DOCX) [file pone.0213294.s006.docx]
