# Supplementary material for: Mesothelin and TGF-α predict pancreatic cancer cell sensitivity to EGFR inhibitors and effective combination treatment with trametinib
Source: PLoS One. 2019 Mar 28;14(3):e0213294. doi: 10.1371/journal.pone.0213294 (PMC6438513; doi:10.1371/journal.pone.0213294)

**S7 Fig:** Combination treatment of cetuximab and gemcitabine in select cell lines. MTT of 6-day treatment of the 100 nM cetuximab alone or in combination with 100 nM or 1 µM gemcitabine in (A) MIA-PACA, (B) PANC-1, (C) CFPAC-1, (D) HPAF-II, (E) PL45, and (F) CAPAN-2 cells. * denotes p <0.05 when compared to control by one-way ANOVA and Tukey post-test. # denotes p <0.05 when compared to 100 nM cetuximab alone and 100 nM gemcitabine alone by one-way ANOVA, Tukey post-test , and Chou Talalay CI values equal to or less than 1. & denotes *p* <0.05 when compared to 100 nM cetuximab alone and 1 µM gemcitabine alone by one-way ANOVA, Tukey post-test, and Chou Talalay CI values equal to or less than 1. Assays were completed in triplicate.


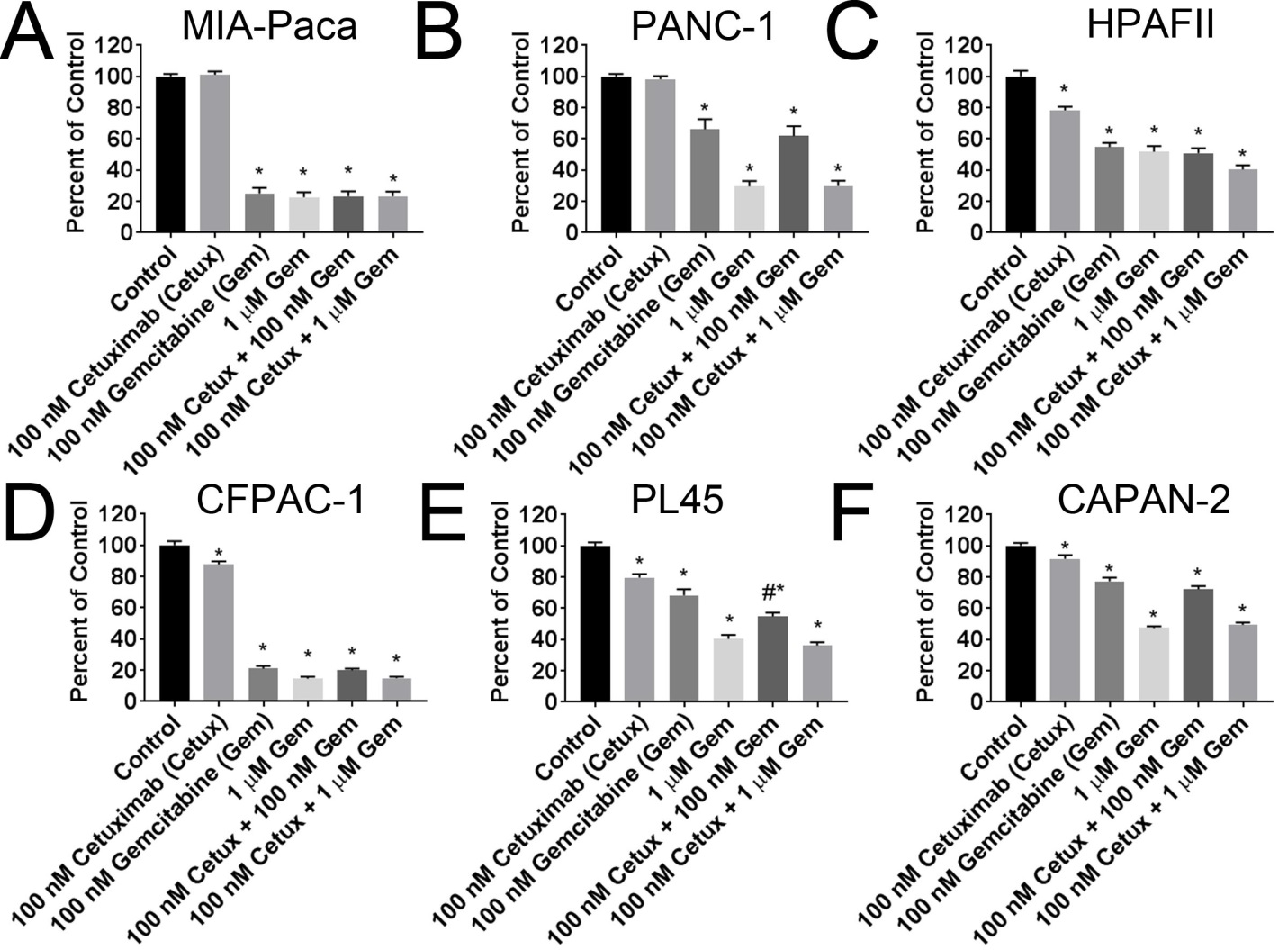

Supplement: S7 Fig — MTT of 6-day treatment of the 100 nM cetuximab alone or in combination with 100 nM or 1 μM gemcitabine in (A) MIA-PACA, (B) PANC-1, (C) CFPAC-1, (D) HPAF-II, (E) PL45, and (F) CAPAN-2 cells. * denotes p <0.05 when compared to control by one-way ANOVA and Tukey post-test. # denotes p <0.05 when compared to 100 nM cetuximab alone and 100 nM gemcitabine alone by one-way ANOVA, Tukey post-test, and Chou Talalay CI values equal to or less than 1. & denotes p <0.05 when compared to 100 nM cetuximab alone and 1 μM gemcitabine alone by one-way ANOVA, Tukey post-test, and Chou Talalay CI values equal to or less than 1. Assays were completed in triplicate. (DOCX) [file pone.0213294.s007.docx]
