# Supplementary material for: Mesothelin and TGF-α predict pancreatic cancer cell sensitivity to EGFR inhibitors and effective combination treatment with trametinib
Source: PLoS One. 2019 Mar 28;14(3):e0213294. doi: 10.1371/journal.pone.0213294 (PMC6438513; doi:10.1371/journal.pone.0213294)
Supplement: S1 Table — (DOCX) [file pone.0213294.s009.docx]

S1 Table: List of antibodies used in this study
