# Supplementary material for: Mesothelin and TGF-α predict pancreatic cancer cell sensitivity to EGFR inhibitors and effective combination treatment with trametinib
Source: PLoS One. 2019 Mar 28;14(3):e0213294. doi: 10.1371/journal.pone.0213294 (PMC6438513; doi:10.1371/journal.pone.0213294)
Supplement: S3 Table — (DOCX) [file pone.0213294.s011.docx]

S3 Table: Correlation of gefitinib sensitivity to the indicated proteins
